# Recent Advances, Challenges, and Functional Applications of Protein Chemical Modification in the Food Industry

**DOI:** 10.3390/foods14162784

**Published:** 2025-08-10

**Authors:** Peiming Zhao, Zhiyan Zhang, Wei Ran, Ting Bai, Jie Cheng, Jiamin Zhang

**Affiliations:** 1 Meat Processing Key Laboratory of Sichuan Province, College of Food and Biological Engineering, Chengdu University, Chengdu 610106, China; 2 Cuisine Science Key Laboratory of Sichuan Province, Sichuan Tourism University, Chengdu 610100, China; 3 Sichuan Provincial Engineering Research Center of Meat Quality Improvement and Safety Control Technology, College of Food and Biological Engineering, Chengdu University, Chengdu 610106, China

**Keywords:** protein chemical modification, functional properties, food industry applications, AI-driven modification

## Abstract

Proteins serve as crucial functional components in food processing, with their unique physicochemical properties directly influencing the texture and stability of food products. Proteins exhibit a range of functional properties, including emulsification, foaming, gelation, and hydration. These properties arise from the structural differences in protein molecules. To equip proteins with enhanced and diversified biological functions, researchers have developed a variety of protein modification techniques. Recent breakthroughs in artificial intelligence technologies have opened new opportunities for research on protein chemical modifications. Novel algorithms based on advanced techniques, such as deep learning, image recognition, and natural language processing, have been developed for intelligent prediction of protein modification sites. The application of these AI technologies provides innovative research tools and methodological support for rational design and targeted engineering of protein functions. This review delves into the applications of chemical modification methods aimed at improving protein solubility, emulsifying capabilities, gelation capacity, antioxidant activity, antimicrobial properties, and nutritional value. These modifications alter the structural and functional attributes of proteins, significantly enhancing their performance within food systems and expanding their application prospects in such domains as medicine and biomaterials.

## 1. Introduction

Proteins, which are complex biomolecules, primarily consist of amino acids and elements including carbon, hydrogen, oxygen, nitrogen, and sulfur. As crucial nutritional components in food, proteins not only provide essential amino acids but also play a key role in maintaining cellular structure and function [1,2]. Additionally, proteins exhibit functional properties, such as thickening, gelation, emulsification, structuring, water binding, adhesion, and cohesion [3], which significantly influence the texture, flavor, and processing characteristics of foods. These functionalities enrich the nutritional profile of foods [4,5], enhance sensory properties [6], enhance antioxidant capacity [6,7,8], and include technical roles, such as acting as packaging matrices [9].

**Figure 1:**
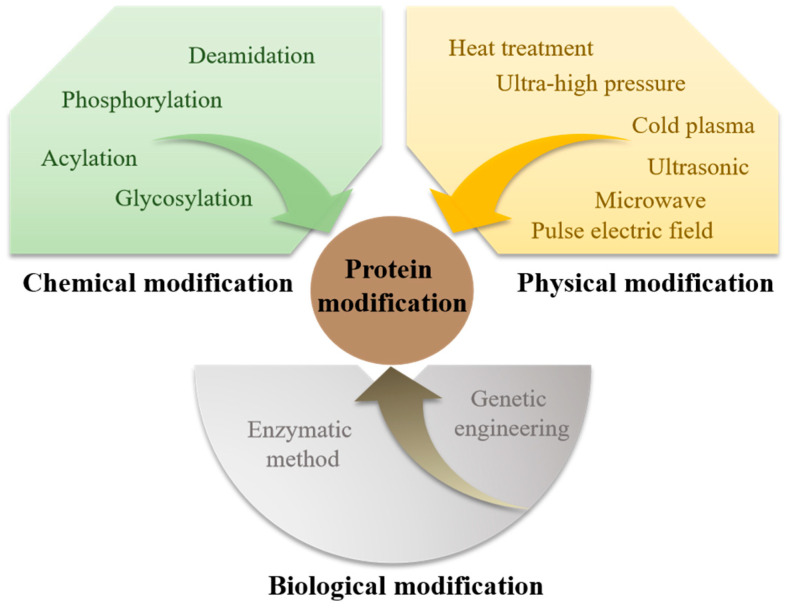
The important modification methods for proteins include chemical modification, physical modification, and biological modification.

However, the inherent structure of proteins often presents challenges in food processing and applications, particularly with plant proteins, which can impart undesirable flavors, such as beany, grassy, or bitter notes, adversely affecting the overall flavor profile of foods [10,11]. Plant proteins typically possess suboptimal solubility and emulsifying properties, restricting their application in food processing [12]. Moreover, the allergenic potential of proteins, such as those derived from milk, soy, and peas, poses health risks for certain consumers [13,14,15], while muscle proteins are vulnerable to enzymatic hydrolysis and oxidation, processes that can diminish functionality [16]. Furthermore, high-temperature exposure induces denaturation, which significantly impairs the emulsifying and foaming properties of proteins [17]. To address these challenges, a variety of chemical protein modification techniques have been developed to enhance desired functional properties and optimize processing outcomes, thus broadening their applications in the food industry [18,19]. Figure 1 shows the main methods of protein modification.

As our understanding of protein functions deepens, protein modification is increasingly being utilized in such fields as food, agriculture, and biomedicine [20,21,22,23]. In the food industry, protein modification is employed to control various properties, such as the water-holding capacity in meat products [24], emulsion stability [25], and gelation [26]. Wang et al. [27] discovered that complexation of γ-cyclodextrin with epigallocatechin-3-gallate (EGCG) ester and myofibrillar proteins (MPs) through intermolecular interactions and disulfide bonds enhances the integrity of gel networks, thereby improving the water-holding capacity and texture of minced shrimp and fish products. Qin et al. [28] employed ultrasonic treatment combined with transglutaminase enzyme cross-linking to enhance the molecular structure and intermolecular cross-linking of wheat gluten proteins, significantly improving their gelation properties. Chemical modification can either preserve or enhance nutritional value, Gerrard et al. [29] demonstrated that acylation enhances the digestibility and absorption rate of egg white proteins (EWPs). In the field of biomedicine, biotechnology companies are developing protein-based pharmaceuticals [30,31], functional foods, and biosensors, where protein modification techniques play a crucial role, offering numerous possibilities for pharmaceutical and food enterprises.

This review systematically examines chemical protein modification techniques and their role in enhancing the functional properties and processing effects of proteins. Additionally, it addresses the challenges and future prospects of developing novel foods through protein modifications, thereby providing deeper insights into this process and fostering ongoing innovation in the food industry.

## 2. Types and Methods of Protein Chemical Modification

Chemical modification involves introducing chemical modifiers that target hydroxyl, amino, and carboxyl groups in proteins or altering the pH value of the system, resulting in changes to protein polypeptide chains and amino acid residues. These changes result in alterations to the spatial structure, hydrophobic groups, and electrostatic charges of proteins, ultimately enhancing their functional properties [32]. Protein chemical modification techniques, like glycosylation, phosphorylation, covalent cross-linking, deamidation, and acylation, offer advantages, including low cost, brief reaction times, straightforward control over the reaction extent, significant effects, and widespread applications [33]. The pathways of protein chemical modification methods are summarized in Table 1.

### 2.1. Deamidation

The deamidation mechanism in proteins involves the hydrolysis of asparagine and glutamine residues, leading to the removal of amide groups and the formation of aspartic acid, glutamic acid, and an ammonia molecule [44]. The removal of amide groups from proteins enhances their solubility, emulsification, and foaming properties. Liao et al. [34] demonstrated that deamidation using acetic acid effectively increases the charge density and electrostatic repulsion of wheat gluten proteins, promoting protein unfolding. This results in reduced protein molecular weight, net charge, and surface hydrophobicity, thereby enhancing protein solubility and emulsification properties. Wang et al. [45] applied protein glutaminase for beef deamidation, finding that deamidation levels of 5% or 7% initiated myofibril dissociation without significantly affecting MP hydrolysis, thus tenderizing meat while preserving water-holding capacity. Shi et al. [35] used natural deep eutectic solvents for rice protein (RP) deamidation, modifying charge density and converting hydrophobic amide groups into hydrophilic carboxyl groups, thereby enhancing solubility, emulsification stability, and activity. Yang et al. [36] utilized a combination of phosphorylation, disulfide bond reduction, and deamidation to enhance RP solubility from 1.29% to 51.45%.

### 2.2. Phosphorylation

**Figure 2:**
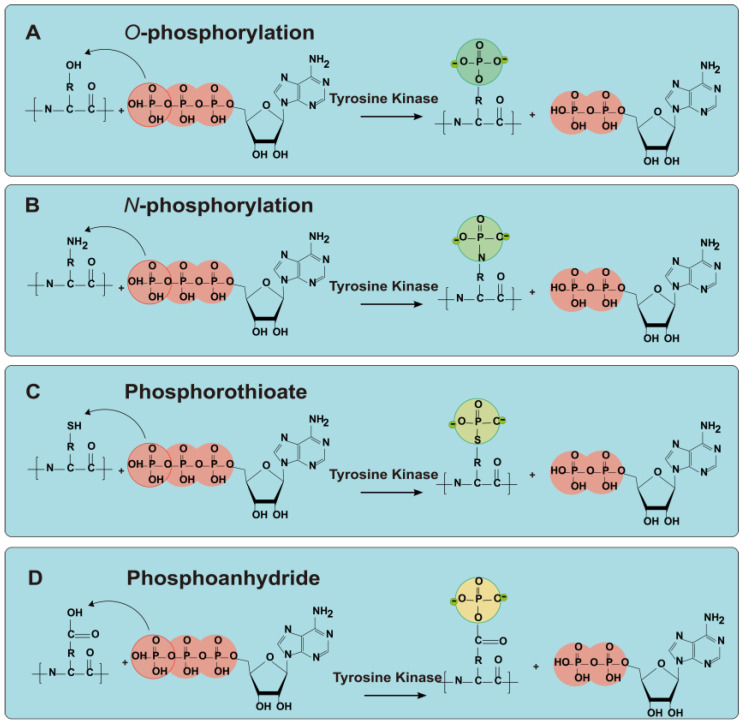
Schematic representation of protein phosphorylation pathways. (**A**) The process of *O*-phosphorylation (pSer, pThr, pTyr); (**B**) The process of *N*-phosphorylation (pHis, pArg, pLys); (**C**) The process of Phosphorothioate (pCys); (**D**) The process of Phosphoanhydride (pAsp, pGlu).

Protein phosphorylation occurs through two pathways, namely enzymatic and non-enzymatic methods. Non-enzymatic phosphorylation involves an esterification reaction between active groups (-OH, -NH_2_) on protein side chains and phosphates, which is catalyzed by phosphorylating agents that introduce numerous phosphate groups [46]. Figure 2 represents the pathways of protein phosphorylation. This reaction enhances the electronegativity and intermolecular electrostatic repulsion among protein molecules, thereby improving their dispersibility and processing characteristics, such as emulsification, foaming, and solubility [47]. Campbell et al. [38] conducted phosphorylation on soy protein isolate (SPI), resulting in significant improvements in solubility, emulsification, and foaming properties. Nayak et al. [48] added POCl_3_ to buffalo milk proteins (casein, precipitates, and whey proteins) for phosphorylation, which cross-linked hydrophilic phosphate groups to the protein side chains and significantly enhanced the solubility of whey proteins in aqueous, Na salt, and Ca salt solutions. Zhao et al. [37] examined the effects of various concentrations of sodium trimetaphosphate (STMP) and sodium tripolyphosphate on perilla protein isolate, noting that phosphorylation increased particle size and electronegativity, exposed hydrophobic and free groups, and enhanced protein solubility to 92.87% at a 6% STMP concentration. Sanchez-Resendiz et al. [49] performed phosphorylation on SPI and peanut protein isolates (PPI), recording increases in emulsifying activity (soy by 27.3%, peanut by 6.6%) and a 1.5% increase in in vitro digestibility, suggesting that these modified isolates could serve as effective substitutes for producing innovative and nutritious food components.

### 2.3. Glycosylation

Protein glycosylation types are categorized based on the glycosidic donor and the nature of protein binding, either covalent or non-covalent [50]. Non-enzymatic glycosylation involves the formation of glycosylated proteins through the covalent linkage of sugars to the carboxyl or amino groups of proteins [51]. Glycosylation can influence both the stability and activity of proteins, thereby effectively enhancing their functional properties [52,53]. Chobert et al. [41] introduced various sugars, including arabinose and ribose, to casein, and following the Maillard reaction, these sugars facilitated the formation of stable covalent bond polymers, thus enhancing the foaming and emulsifying properties of casein. Naotoshi et al. [39] used galactomannan to glycosylate EWP, noting that the modified proteins exhibited improved gel strength and water-holding capacity compared to the control group. Jimenez-Castano et al. [54] observed enhanced thermal stability in β-lactoglobulin following its glycosylation with dextran at pH 5 and 85 °C. Mu et al. [55] prepared a soy protein–arabic gum copolymer via a wet-heat Maillard reaction, which increased the solubility under isoelectric conditions by nearly 40%. Nakamura et al. [24] used galactomannan to glycosylate EWP and lysozyme, finding that the emulsifying activity of the EWP increased by 15%, and the lysozyme-galactomannan conjugate demonstrated significant antibacterial effects. Dan et al. [56] prepared whey protein isolate (WPI)–dextran covalent complexes via a wet-heat glycosylation method, finding that these complexes showed enhanced adsorption capacity on oil droplets, significantly improving the emulsifying properties of the WPI.

### 2.4. Acylation

Protein acylation involves the reaction between nucleophilic groups of proteins and acylating agents, introducing new functional groups into the proteins. Following acylation, the surface negative charge of proteins increases, polypeptide chains extend, and significant spatial structural changes occur, thereby lowering the isoelectric point. Enhanced solubility of proteins in weakly acidic, neutral, and alkaline solutions alters their physicochemical properties and functionalities including their emulsifying properties, emulsion stability, and hydrophobicity [57]. Shen et al. [58] conducted a combined modification of pea protein isolates through acetylation and association with guar gum, observing significant increases in oil and water-holding capacities, at 2.09 g oil/g protein and 7.01 g water/g protein, respectively. The modified proteins exhibited significant enhancements in emulsifying capacity and stability, achieving 96% and 95%, respectively. Mirmoghtadaie et al. [42] discovered that succinylation increased the negative charge of oat isolate proteins, enhancing the repulsion between proteins and hindering their interactions, thereby affecting emulsion stability. Zhang et al. [59] conducted dual modifications on ovalbumin through succinylation and ultrasonication, and the modified protein emulsion exhibited characteristics, such as smaller particle size (243.33 nm), reduced viscosity, and weakened gelation, resulting in a 2.7-fold increase in emulsifying capacity and a 7.3-fold increase in emulsion stability. Lang et al. [43] examined the effects of succinylation on MP; with the degree of acylation increasing from 1.46% to 94.99%, the net surface charge and solubility of the protein rose by 2.71 and 13.50 times, respectively, while emulsifying activity and stability improved by 2.32 and 1.46 times, respectively, effectively broadening the application of MP in the food industry.

### 2.5. Other Chemical Modifications

In addition to the previously mentioned chemical modification methods, other techniques, such as cationization, metal ion substitution, and pH-shifting, can also regulate protein structure and functionality [60]. Some examples of chemically modified proteins in recent years are listed in Table 2. These methods enhance antioxidative properties, solubility, bioactivity, and molecular interactions of proteins by either introducing or modifying specific chemical groups within these molecules [61]. Nesterenko et al. [62] studied the encapsulation of vitamins in SPI and found that cationization, which attaches quaternary ammonium cationic groups to the protein chains, enhanced the solubility of the modified SPI, thus facilitating the microencapsulation of ascorbic acid. Li et al. [63] employed pH-shifting to modify PPI, noting that at pH 10, the particle size decreased, while the content of free thiol groups, surface hydrophobicity, and solubility increased, significantly enhancing the gel’s breaking force and water-holding capacity. Yan et al. [64] investigated the impact of pH-shifting and EGCG on the functional properties of SPI. Initially, they treated SPI under alkaline conditions (pH 12) to unfold the protein structure and subsequently refolded it under neutral conditions. They discovered that the alkaline treatment enhanced the protein’s solubility by 34% and increased the surface hydrophobicity approximately threefold. Zhao et al. [65] studied the impact of metal ions on the fiber formation, structure, morphology, and gel properties of soy protein isolate fibers, discovering that the addition of Ca or Mg led to a more ordered structure, elevated β-sheet content, and enhanced fiberization of SPI, inducing the rapid formation of high-viscosity gels. These modification techniques provide a rich toolkit for the functional regulation of proteins and food processing.

## 3. The Improvement of Protein Functional Properties Through Chemical Modification

### 3.1. The Improvement of Protein Solubility Through Chemical Modification

**Figure 3:**
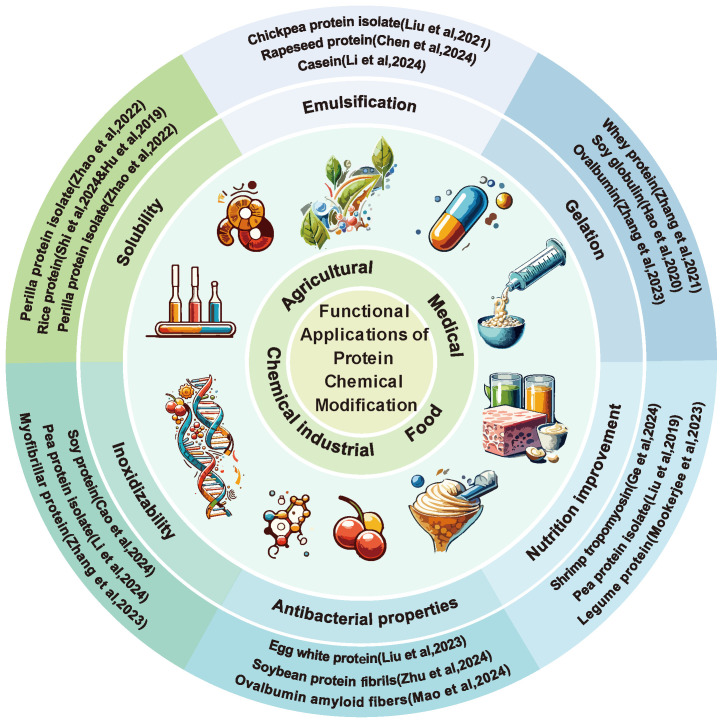
The impact of chemical modification on the functional properties of proteins [37,40,59,70,77,78,79,80,81,82,83,84].

Protein solubility, defined as the degree to which proteins dissolve in solvents, is influenced by various factors, such as pH, temperature, ionic strength, and solvent properties [73]. Proteins possess hydrophobic and hydrophilic regions; the hydrophobic regions typically cluster together, forming the core structure of the protein, whereas the hydrophilic regions interact with water molecules in the solvent. Chemical modification can improve hydrophilicity at the nano- and microstructural levels by introducing strongly hydrophilic groups on the surface of proteins; strong interactions between the hydrophilic regions of the protein and the water molecules in the solvent generally lead to increased solubility [74]. The impact of chemical modification on the functional properties of proteins are illustrated in Figure 3. Hadidi et al. [75] employed microwave-assisted phosphorylation to modify mung bean protein, thereby enhancing the electrostatic repulsion between protein molecules and their dispersion in solution systems, which, in turn, increased the solubility and broadened its applications in pharmaceutical and food products. Miedzianka et al. [76] performed acetylation on RP concentrate, significantly improving its water-binding capacity and solubility, thereby paving the way for its use in a variety of food products, including meat, fish, milk, frozen desserts, and as a coffee creamer. Hu et al. [67] used STMP for the thermal phosphorylation of rice bran protein (RP), demonstrating that at pH 9, the solubility of the modified RP peaked at 58.4%. Analyses using Fourier transform infrared spectroscopy and X-ray photoelectron spectroscopy confirmed the formation of phosphate esters (P = O), indicating that phosphorylation can significantly enhance the functional properties of RP.

### 3.2. The Improvement of Protein Emulsification Through Chemical Modification

The emulsifying capacity of proteins is defined by their ability to stabilize emulsions under appropriate conditions [85]. Proteins, which inherently possess hydrophilic and lipophilic groups, function similarly to nonionic surfactants, primarily serving to thicken and emulsify fats [86]. The emulsifying capacity of proteins is influenced by various factors, such as molecular structure, charge properties, physicochemical properties, and protein components [87]. Protein molecules typically exhibit complex structures, including α-helix, β-sheet, and random coil configurations. After chemical modification, proteins can exhibit improved conformational flexibility and stability to form dense interfacial films. Through the mechanical strength, steric hindrance, and electrostatic repulsion of the interfacial film, their interfacial behavior and emulsion stability are optimized, ultimately enhancing their emulsifying properties [88]. The emulsifying properties of proteins are crucial in the production of various foods, such as sausages, cakes, and ice cream. Li et al. [89] employed the Maillard reaction to modify natural agar, thereby enhancing the emulsifying performance of the resulting covalent products, which were subsequently applied as stabilizers in mayonnaise preparation, offering novel insights into mayonnaise development. Yan et al. [90] utilized the Maillard reaction with dextran grafting to modify PPI and found that the proportion of β-sheet in the dextran-protein conjugate increased, affecting the protein’s secondary structure and enhancing the emulsifying activity index (EAI) by 136%. Tan et al. [91] investigated the effects of high hydrostatic pressure (HHP) combined with pH-shifting on the structure and emulsifying properties of SPI, finding that the synergistic action of HHP and pH-shifting significantly unfolded the protein structure, induced the formation of smaller reaggregated protein particles, and enhanced the protein’s emulsifying performance.

### 3.3. The Improvement of Protein Gelation Through Chemical Modification

Protein gels form as three-dimensional network structures, either solid or semi-solid, in which proteins in a solution entrap solvents [92,93]. The gelation properties of proteins are determined by their molecular structure and interactions. Chemical modification of proteins regulates intermolecular interactions and aggregation behavior by altering molecular conformation at the nanoscale, surface properties, and functional group composition, thereby optimizing the microgel network structure [94,95]. These gels absorb moisture and flavor substances, thereby stabilizing a uniform mixture of water, fat, protein, starch, and other components in gel-based foods [92]. By utilizing these properties, the texture, sensory characteristics, and structural properties of protein gel foods can be fine-tuned [96,97]. The gelation properties of proteins are widely utilized in food products and serve as a critical index in food applications [98]. Hao et al. [40] modified soybean globulin using water-soluble polysaccharides, thereby improving the structural stability of soybean globulin in secondary and tertiary structures; consequently, the modified emulsion exhibited enhanced gelation and thermal stability. Zhang et al. [77] prepared whey protein–flaxseed gum conjugates through both traditional and ultrasound-assisted Maillard reactions to encapsulate astaxanthin. The study demonstrated that the emulsion gel’s stability was enhanced, and the bioavailability of astaxanthin reached 72.08%. Spotti et al. [99] observed that the Maillard reaction significantly reduced the free thiol group content in whey protein from 24.37 µmol SH/g to 12.70 µmol SH/g, a change attributed to the consumption of thiol groups during the reaction. This not only affected the interactions between protein molecules but also altered the mechanism of gel network formation.

### 3.4. The Improvement of Protein Inoxidizability Through Chemical Modification

Chemical modification of proteins enhances their ability to scavenge free radicals or inhibit oxidant activity by exposing hidden antioxidant groups through conformational unfolding, introducing new active groups, or optimizing charge distribution, thereby enhancing metal chelating ability [78,100,101]. In food processing, antioxidant-modified proteins can be integrated into various products to decrease the reliance on synthetic antioxidants, thereby preserving the freshness and nutritional value of foods [102,103], enhancing food stability, and meeting consumer demand for healthy and safe options [104]. Li et al. [79] prepared a ternary complex of pea protein isolate, EGCG, and iron ions (Fe^3+^), demonstrating that this complex exhibited a DPPH radical scavenging activity of over 90%, indicative of strong antioxidant activity. Additionally, the complex significantly hindered the growth of *Escherichia coli* and *Staphylococcus aureus*, illustrating its potential as a food preservative and nutritional enhancer. Zhang et al. [80] studied the effects of water extracts from red peppers and garlic on the antioxidant capacity of MP and found that the antioxidant components effectively inhibited the formation of carbonyl compounds and cross-linked polymers during protein oxidation. Yan et al. [105] investigated the protein structural characteristics and antioxidant activity of the protein isolate (PI) from *Cinnamomum camphora* seed kernel and purified phenolic extracts (PPEs) after covalent modification, finding that the secondary and tertiary structures of the PI-PPE complex became more ordered, and the DPPH and ABTS radical scavenging activities of the PI-PPE complex increased by 42% and 34%, respectively.

### 3.5. The Improvement of Protein Antibacterial Properties Through Chemical Modification

The application of protein modification for antimicrobial properties involves adjusting protein molecular structures to impart antimicrobial characteristics [106]. Chemical modification of proteins enhances their electrostatic attraction with microorganisms and membrane insertion ability by regulating the surface charge of proteins and conjugating antibacterial functional groups, thereby strengthening their interaction with microbial cell walls to disrupt cell membranes, inhibiting metabolic activity, or directly inducing microbial death [107]. Research and application of these properties are aimed at controlling microbial growth and reproduction, thus reducing the risk of infections and diseases [108]. In food processing, protein modification can be employed to develop antimicrobial packaging materials and food additives, thereby extending the shelf life of food and reducing the risk of food poisoning [81,109]. Zhu et al. [82] investigated the interaction mechanism between soy protein protofiber (PF) and chlorogenic acid (CA), with a focus on its antimicrobial activity. When the CA/PF ratio exceeded 0.05, the characteristic structure of the protofiber was disrupted, resulting in the PF becoming shorter, more flexible, and thicker. This demonstrated that the CA-PF complex could inhibit the growth of *Escherichia coli* and *Staphylococcus aureus*. Mao et al. [81] explored the characteristics of a hydrogel formed by ovalbumin amyloid fibers (OAFs) induced by EGCG. As the concentration of EGCG increased, the diameter of the OAFs increased, the content of β-sheet structures rose, and the hydrogel exhibited good viscoelasticity and thermal stability, Additionally, it showed strong antimicrobial activity against both Gram-negative and Gram-positive bacteria, suggesting potential applications in antimicrobial food packaging. Liu et al. [83] found that biodegradable composite films made from tea polyphenols and EWP exhibited significant inhibitory effects on *Escherichia coli* and *Staphylococcus aureus*, These films are suitable for preserving refrigerated meats, thereby helping to reduce food waste and enhance food safety.

### 3.6. The Improvement of Protein Nutrition Through Chemical Modification

The nutritional value of proteins is assessed based on such factors as essential amino acid content, protein biological value, and protein digestibility. Chemical modification of proteins cleaves macromolecular proteins into small peptide segments via enzymatic or chemical hydrolysis, which improves digestive enzymes’ access and facilitates the release and absorption of amino acids. Protein modification enhances the nutritional value of food by eliminating antinutritional factors, improving digestibility, enriching flavor, and reducing allergenicity [84,110]. Additionally, modified proteins can be incorporated into various foods as nutritional enhancers to boost their nutritional value and meet the dietary requirements of specific groups [111,112]. Gerrard et al. [29] demonstrated that chemical cross-linking effectively protects lysine from degradation during the Maillard reaction, thereby preserving the nutritional value of proteins. They also treated EWP with glutaraldehyde and glyoxal to acylate it, transforming the microstructure into an open network that improves digestibility. Liu et al. [70] enhanced the EAI of pea protein isolate by 63.07%, the foaming capacity (FC) by 114.28%, and the oil absorption capacity (OAC) by 73.31% through phosphorylation modification. They further explored the potential of modified pea protein isolate to replace cream in mango mousse cakes, demonstrating its versatility in food applications.

## 4. The Effects of Chemical Modification Combined with Other Modification Methods on the Functional Properties of Proteins

### 4.1. Chemical Modification Combined with Physical Modification

The core mechanism of the synergistic modification strategy combining protein chemical and physical modification lies in using physical modification to disrupt the higher-order structure of proteins, exposing originally buried reactive amino acid residues [113]. This provides more accessible sites for subsequent chemical modifications (e.g., acylation, phosphorylation, cross-linking reactions), forming a synergistic effect of multi-scale structure regulation. This synergy significantly enhances protein functional properties, demonstrating broad application value in the food industry—such as improving key functionalities, like emulsifying stability, gel strength, solubility, and digestibility [114]. Jiang et al. [115] modified duck myofibrillar proteins (MPs) with varying concentrations of sodium bicarbonate under cold plasma treatment (50 V, 3 min). The results showed that the addition of sodium bicarbonate increased the pH of duck MPs (from 6.35 to 7.15), reduced turbidity and average particle size (from 1228 nm to 839.5 nm), increased the degree of protein unfolding, and enhanced solubility from 29.85% to 60.73%, thereby enhancing the EAI and emulsifying stability index (ESI). Alavi et al. [116] modified faba bean protein (FPI) using ultrasound-assisted alkaline treatment. The results showed that modified FPI solubility significantly improved, exceeding 95% at both pH 3 and 7. Meanwhile, foaming capacity increased to 306–386%, and foam stability significantly extended from the original 9 s to 974 s. These functional property optimizations greatly expand the application potential of chickpea protein isolate in the food industry.

### 4.2. Chemical Modification Combined with Biological Modification

The synergism of chemical and biological modification demonstrates remarkable advantages in improving protein functional properties by integrating the high selectivity of enzymatic catalysis with the efficiency of chemical modification [117]. Enzymatic modification allows for the regulation of protein spatial structure and functional groups under mild conditions [118], whereas chemical modifications (e.g., acylation, phosphorylation, or glycosylation) can further introduce functional groups or enhance intermolecular interactions [119]. The synergistic effect of the two can be achieved through multiple mechanisms. For instance, enzymatic pretreatment can disrupt the compact protein structure, exposing more active sites to enhance the modification efficiency of subsequent chemical reagents [120]. Conversely, chemical modification may alter the charge distribution or hydrophobicity of proteins, thus optimizing the working environment for enzymes [121]. He et al. [122] performed conjugate modification of zein via trypsin-limited hydrolysis, transglutaminase catalysis, and chitosan oligosaccharide lactate (COL). The results showed that the solubility of modified zein at pH 5, 6, and 7 increased by 72.93%, 72.42%, and 74.17%, respectively. The EAI and ESI improved by approximately 2-fold. The foaming capacity and stability were significantly better than those of unmodified zein and soy protein isolate (SPI). Liu et al. [123] used an enzymolysis–phosphorylation synergistic modification method for porcine hemoglobin (PHb). The results showed that enzymolysis–phosphorylation modification (HP-PHb) decreased the surface hydrophobicity of PHb and significantly improved its solubility. The EAI increased from 43.39 m^2^/g to 55.81 m^2^/g, and the emulsifying stability increased from 55.14% to 61.51%. The thermal denaturation temperature rose from 66.57 °C to 86.57 °C. After adding HP-PHb, the pH of minced meat increased from 6.0 to 6.2, the water-holding capacity was enhanced, and the cooking loss decreased from 38% to 34%. The addition of HP-PHb also improved the texture properties of minced meat, giving it higher elasticity and a denser fibrous structure.

## 5. Artificial Intelligence Empowers the Chemical Modification of Proteins

In recent years, with the rapid development of cutting-edge artificial intelligence (AI) technologies, such as deep learning, image recognition, and natural language processing, machine learning methods based on big data have made remarkable progress in numerous AI fields and have also been widely applied to the research on protein chemical modification [124,125,126]. In the field of protein chemical modification, AI technologies have developed new algorithms for predicting important modification sites by systematically screening the modification sites of important protein functions. By integrating the sample learning and logical reasoning abilities of language models, the functional semantics of chemical modifications have been decoded [127,128,129]. Through the feature encoding of multiple aspects, such as protein sequences, physicochemical properties, and structures at all levels [130], a series of algorithms for predicting protein modification sites based on machine learning or deep learning have been developed [131].

Commonly used feature encodings of protein sequences include pseudo-amino acid composition [132], composition of k-spaced amino acid pairs [133], orthogonal binary coding, amino acid index [134], autocorrelation function, group-based prediction system [135], and position-specific scoring matrix [136], etc. Phosphorylation, acylation (such as acetylation and succinylation), glycosylation, and lipid modifications (such as palmitoylation, myristoylation, geranylgeranylation, and cholesterylation) of specific amino acid residues in proteins can dynamically change the conformation of proteins, thereby affecting their functional properties [137]. Currently, a variety of computational tools have been developed for predicting phosphorylation, acylation, and palmitoylation sites based on protein sequence and structural features [138]. These tools include CSS-Palm [139], GPS-SUMO [140], GPS-PBS [141], GPS-Palm [142], GPS-pPLM [143], SEMal [144], MRMD-palm [145], PWMs, SVM [146], PalmPred [147], SeqPalm, GPS-Lipid [148], HybridSucc [149], and MDD-Palm [150]. Li et al. [151] established a high-accuracy random forest machine learning model for predicting protein acetylation by constructing a species-specific high-precision protein acetylation dataset, extracting various effective features, combined with an efficient feature selection method. They also developed online and local prediction tools for the acetylation site prediction models of different species. The successful development of this bioinformatics tool is of great significance for the in-depth study of the sequence–structure–function relationship of protein acetylation sites. Biggar et al. [152] developed an approach combining in silico prediction with targeted mass spectrometry (MS) to identify Lys methylation (Kme) sites at the proteome level. They created MethylSight, a program that predicts Kme events based on the physicochemical properties of residues around putative methylation sites, which was then validated by targeted MS. GO analysis and SAFE analysis of the predicted methyllysine proteome indicated significant enrichment in various cellular processes, such as ribosome biogenesis, protein translation, and DNA metabolism. This study is of great significance for further research on lysine methylation.

## 6. Conclusions

After modification, proteins can enhance the nutritional value, taste, and storage stability of food, offering extensive applications in the food industry. These enhancements include functional improvements, nutritional enhancements, and antimicrobial properties, among others. These applications enhance food quality and functionality, addressing consumer demand for health, nutrition, and convenience, and driving the continuous development of the food industry.

Currently, protein modification methods face limitations, including unstable modification effects, complex processes, and elevated levels of pollution. Some protein modification methods may pose safety risks, including the generation of byproducts or residual substances. Consequently, future research will focus on exploring innovative modification methods and technologies to enhance the safety and sustainability of these processes. Innovations in methods and technologies, including genetic engineering and the use of auxiliary agents, are being developed to achieve more precise and efficient protein modifications, further enhancing food quality, functionality, and safety. This will introduce new opportunities and challenges in the food industry, promoting texture-optimized and healthier food while addressing consumer demand for food safety and environmental sustainability.

## Figures and Tables

**Table 1:** A summary of the chemical modification methods of proteins.

Modification Method	Protein Type	Strategy	Critical Operational Step	Modified Effect	Bottlenecks	References
Deamidation	Wheat gluten; rice protein	Acetic acid (0.03–0.14 mol/L) and HCl (0.05–0.22 mol/L) were added separately to a 100 g/L wheat gluten suspension and heated at 121 °C.	Acid/enzyme concentration	Disrupts H-bonding; promotes backbone cleavage (Asn), enhancing the charge density and electrostatic repulsion of proteins and improving emulsification, emulsion stability, and solubility	Unstable modification efficiency	[34,35,36]
Phosphorylation	Perilla protein isolate; soy protein isolate	PPI was mixed with STPP and STMP, adjusted to pH 9, and agitated at 45 °C for 2 h.	Phosphorylating agent selection and dosage, pH regulation	Induces conformational shifts; alters electrostatic interactions, enhancing the electrostatic repulsion between protein molecules; results in improved solubility, emulsifying properties, and foaming ability	Reagent residue in final product, requiring additional purification steps	[37,38]
Glycosylation	Egg white protein; soybean globulin; casein	The mixture of GM and DEW, with a weight ratio of 1:4, was subjected to dry-heat treatment at 60 °C and 65% relative humidity.	Dry-heat duration/temperature	Adds hydrophilic glycans; significantly alters hydrophilicity; the mechanical strength and water retention ability of the gel were augmented, concurrently enhancing the gel’s transparency and thermal stability	Undesirable flavor compounds	[39,40,41]
Acylation	Oat protein isolate; myofibrillar proteins	Add succinic anhydride to an OPI aqueous suspension that has a pH of 8 and a concentration of 5%.	pH regulation and acylating agent dosage	Adds hydrophobic chains, significantly enhance the solubility and emulsifying properties of the protein	Unreacted acylating agents may remain, raising food safety concerns	[42,43]

**Table 2:** A summary of the impacts of chemical modification approaches on the functional properties of proteins.

Modification Method	Protein Type	Chemical Reagents	Functional Characteristics	References
Phosphorylation	Soybean protein isolate; buffalo milk proteins	Sodium tripolyphosphate and sodium hexametaphosphate	Structural changes, emulsibility, solubility	[48,66]
Acylation	Pea protein isolate; egg white proteins	Acetic anhydride and succinic anhydride	Oil-holding capacity, gelation, emulsibility	[29,58]
Phosphorylation	Rice bran protein	Sodium trimetaphosphate	Structural changes, solubility, emulsibility activity, solubility	[67]
Glycosylation	Sesame protein	Gum arabic	Solubility, thermal stability	[68]
Phosphorylation, succinylation, deamidation, and glycosylation	Silkworm pupae proteins	Sodium tripolyphosphate, succinic anhydride, and acetic acid	Water-holding capacity, foaming ability and foaming stability	[69]
Phosphorylation	Pea protein isolate	Sodium tripolyphosphate	Solubility, viscosity, emulsibility, foaming ability	[70]
Deamidation	Wheat gluten; beef myofibrillar proteins	Acetic acid, tartaric acid, and citric acid	Allergenicity, water-holding capacity, emulsibility, solubility	[45,71]
Glycosylation	Black rice glutelin; rabbit myofibrillar proteins	Arabinose, sodium alginate, maltodextrin, and lactose	Structural changes, solubility, emulsion stability	[50,72]

## Data Availability

No new data were created or analyzed in this study. Data sharing is not applicable to this article.
